# Pharmacokinetics and bioequivalence of two formulations of the S-1 (tegafur/gimeracil/oxonate) capsule in Chinese cancer patients under fasting and fed conditions: a multicenter, randomized, open-label, single-dose, double-cycle crossover study

**DOI:** 10.3389/fphar.2025.1494902

**Published:** 2025-02-19

**Authors:** Junli Lu, Yuyan Lei, Yuanyuan Mo, Xiangping Wang, Wanying Liu, Yu Yan, Hongying Yang, Canxia Li, Lifeng Huang, Qiuxia Shen, Caihong Wang, Jingjie Chen, Lulu Chen, Xiaohui Li

**Affiliations:** ^1^ Phase Ⅰ Clinical Trial Laboratory, The Second Nanning People’s Hospital, Nanning, Guangxi, China; ^2^ Department of Pharmacology, School of Pharmaceutical Science, Central South University, Changsha, Hunan, China; ^3^ Qilu Pharmaceutical Co., Ltd., Jinan, Shandong, China; ^4^ Hunan Key Laboratory for Bioanalysis of Complex Matrix Samples, Changsha, Hunan, China

**Keywords:** S-1, bioequivalence, pharmacokinetics, tegafur, 5-fluorouracil, safety, cancer patients

## Abstract

**Objective:**

S-1, an oral multicomponent capsule containing tegafur, gimeracil, and potassium oxonate, has demonstrated efficacy in various tumor types. This study aimed to assess the pharmacokinetics, bioequivalence (BE), and safety of a newly developed generic S-1 capsule compared to the original brand-name formulation in Chinese cancer patients under fasting and fed conditions.

**Methods:**

A multicenter, randomized, open-label, single-dose, double-cycle crossover study was conducted in Chinese cancer patients. The study involved 120 subjects, with 60 assigned to the fasting group and another 60 to the fed group. In each study cycle, subjects were randomly assigned toreceive either the reference or test S-1 capsule at a 7-day interval. Blood samples were collected for analysis within 48 h after ingestion. The plasma concentrations of tegafur, 5-fluorouracil, gimeracil, and potassium oxonate were determined by liquid chromatography–tandem mass spectrometry (LC-MS/MS). The main pharmacokinetic (PK) parameters were calculated using the non-compartmental approach. BE was assessed through geometric mean ratios (GMRs) between the two formulations and their respective 90% confidence intervals (CIs). The safety of the two formulations was also evaluated.

**Results:**

The pharmacokinetics of the two formulations were similar under both fasting and fed conditions. The 90% CIs of the GMRs for the maximum observed serum concentration (C_max_), AUC_0-t_, and AUC_0- ∞_ ratios were observed to lie within the BE acceptance range of 80%–125%. Both formulations of the S-1 capsule exhibited similar adverse events (AEs), primarily including decreased white blood cell count and hypertension. These AEs were generally mild and transient. The safety profiles of the two formulations were found to be good and comparable, with no serious adverse events (SAEs) reported.

**Conclusion:**

The newly developed generic S-1 and reference formulations exhibit comparable PK in Chinese cancer patients in the fasting and fed state. The formulations of S-1 showed good tolerability and a similar safety profile.

**Clinical Trial Registration:**

http://www.chinadrugtrials.org.cn/index.html, identifier CTR20171562.

## Introduction

The therapeutic regimen incorporating 5-fluorouracil (5-FU) is globally recognized as the conventional chemotherapy treatment for a myriad of cancer types (Satoh and Sakata, 2012). In order to mitigate the issue of rapid degradation, S-1 was innovated as an advanced fourth-generation oral fluoropyrimidine derivative, which encompasses three pharmacological entities, namely, tegafur (FT), gimeracil (CDHP), and potassium oxonate (OXO), formulated in a molar ratio of 1:0.4:1 (Satoh and Sakata, 2012; Kawahara, 2014).

Among these, FT serves as a prodrug of 5-FU, which undergoes continuous conversion to 5-FU, predominantly facilitated by cytochrome P450 2A6 (CYP2A6) after oral absorption, thereby exerting its anticancer efficacy (Wang et al., 2011). CDHP acts as a competitive inhibitor of the enzyme dihydropyrimidine dehydrogenase (DPD), thereby limiting the degradation of 5-FU and resulting in prolonged, efficacious concentrations of 5-FU in both plasma and tumor tissue (Diasio, 1999; Furuse et al., 2010). The inhibitory effect of CDHP on DPD substantially contributes to reducing 5-FU catabolism, thereby achieving significantly elevated plasma levels of 5-FU compared to the administration of FT alone (Saif et al., 2011). OXO, also known as oteracil, is specifically localized to the gastrointestinal mucosa, where it selectively targets and inhibits orotate phosphoribosyl transferase (OPRT). This targeted inhibition effectively blocks the phosphorylation of 5-FU, thereby reducing the gastrointestinal toxicity commonly linked to 5-FU therapy (Saif et al., 2009). Consequently, S-1 is frequently called a “self-rescuing” therapeutic agent (Zhuang et al., 2013). When administered as a continuous infusion, S-1 demonstrated reduced toxicity and enhanced antitumor efficacy compared to uracil-FT and 5-FU (Hirata et al., 1999; van Groeningen et al., 2000).

Initially approved in Japan in 1999 as a therapeutic intervention for gastric cancer (Koizumi et al., 2000), S-1 has subsequently been validated for its efficacy across a spectrum of malignant tumors, such as breast cancer (Toi et al., 2021), head and neck cancer (Shih et al., 2021), colorectal cancer (Yamada et al., 2018; Miyamoto et al., 2014), lung cancer (Nokihara et al., 2017; Kawahara, 2014), pancreatic cancer (Uesaka et al., 2016), prostate cancer (Akaza et al., 2010), and cholangiocarcinoma (Nakachi et al., 2023). Owing to its superior anticancer properties and minimal toxicity, generic formulations of S-1 have been widely promoted in various countries. Despite its approval for a diverse array of cancer types, pharmacokinetic (PK) studies of S-1 have frequently been limited to individual cancer types. Furthermore, there is a notable scarcity of large-scale comparative studies on the pharmacokinetics of S-1 in both the fasting and fed states (Lee et al., 2019; Zhuang et al., 2013; Chen et al., 2020). In order to rigorously address these identified gaps, we orchestrated a multicenter study aimed at comparing the PK and bioequivalence (BE) of a novel generic S-1 formulation (Qilu Pharmaceutical Co., Ltd., Shandong, China) with an established reference formulation (Taiho Pharmaceutical Co., Ltd., Japan) in a large cohort of 120 Chinese patients, with a variety of cancer types under both fasting and fed conditions.

## Materials and methods

### Ethics and study population

This study was conducted in seven institutions, namely, the First Affiliated Hospital of China Medical University, the Affiliated Cancer Hospital of Harbin Medical University, the First Affiliated Hospital of Nanchang University, the Affiliated Hospital of Qingdao University, the Second People’s Hospital of Nanning, the Guizhou Cancer Hospital, and the Affiliated Hospital of Jiangnan University (China Clinical Trials Registry: CTR20171562). The study protocol and informed consent procedures were approved by the clinical research ethics committees of each participating center. The ethical approval process strictly adhered to the requirements of Good Clinical Practice (GCP), the Declaration of Helsinki, and relevant domestic laws and regulations. Prior to the commencement of the trial, all subjects provided signed informed consent forms, retaining the right to withdraw from the study at any point without the need for explanation or fear of repercussion.

This study enrolled patients aged 18–75 years, who were diagnosed with malignant solid tumors, as confirmed by definitive histopathological and/or cytological diagnoses. The cancer types included head and neck, non-small cell lung, colorectal, breast, and advanced gastric and small bowel cancers, in patients who had not undergone surgical resection. Eligibility was further restricted to individuals with a body mass index (BMI) between 18 and 27 kg/m^2^ and a body surface area (BSA) of at least 1.5 m^2^. Weight criteria specified a minimum of ≥50 kg for male subjects and ≥45 kg for female subjects. The health status of the subjects was assessed through a thorough medical history review, a comprehensive physical examination, and a series of medical investigations, which included routine blood tests, blood chemistry profiles, screening for infectious diseases, and electrocardiograms. The primary exclusion criteria included any abnormalities detected during the physical examination, a significant medical history, or a history of severe drug allergies.

### Study design and drug administration

This study, a multicenter, randomized, open-label, single-dose, two-cycle crossover investigation, was distinctly divided into fasting and fed trials. Each subject received either the test or reference formulation in a separate cycle. Each cycle consisted of a 4-day inpatient phase, followed by a 7-day washout period between cycles to ensure that any residual effects from the previous formulation had fully dissipated before the initiation of the next cycle. To avoid the residual effects of fasting or feeding that could influence the results, the fasting and fed trials each enrolled 60 subjects. Subjects were randomly allocated to one of the two treatment sequences, namely, test-reference or reference-test, in a 1:1 ratio. All eligible subjects were admitted to the clinical ward 1 day prior to drug administration, adhering to a minimum fasting period of 10 h before drug intake. In the fasting trial, subjects were required to fast. Subsequently, they ingested a single dose comprising two capsules (50 mg each) of either the test or reference formulation, accompanied by 240 mL of warm water. In the fed trial, subjects consumed a high-fat, high-calorie meal (protein providing approximately 150 kcal, carbohydrate approximately 250 kcal, fat approximately 500–600 kcal, totaling approximately 800–1000 kcal, with approximately 50% of the calories from fat) within 30 min before orally ingesting a single dose of two capsules (50 mg each) of the test or reference formulation, along with 240 mL of warm water. To verify complete medication intake, the researchers conducted oral examinations of the subjects. Fasting was maintained for 4 h after dosing. Standardized meals were administered 4 and 10 h after dosing.

### Study population

Post-administration of S-1 capsules, the primary constituents identifiable in the body include FT, CDHP, OXO, and the active metabolite 5-FU. Existing research delineates that the AUC_0-t_ of OXO in the organism exhibits the most pronounced coefficient of variation, registering at 34.75% (Lee et al., 2019), thus classifying it as a pharmaceutical with notable pharmacokinetic (PK) variability. The sample size calculation was executed using PASS version 11.0 (NCSS, LLC, located in Kaysville, Utah, United States), based on the following criteria: a within-subject variability of 34.75%, a significance level (α) of 0.05, and a Type II error rate (β) of 0.2. Accounting for a 10% potential dropout rate, the final required number of participants for both the fasting and fed studies was 60 each.

Eligible participants included Chinese cancer patients of both sexes, aged 18–75 years, with body mass indices ranging from 18 to 27 kg/m^2^ and body surface areas of at least 1.5 m^2^. Male participants were required to weigh at least 50 kg, while female participants were required to weigh at least 45 kg. The health status of subjects was meticulously evaluated through various methods, such as medical history assessments, physical examinations, comprehensive medical investigations (encompassing routine hematology, biochemistry panels, and screenings for infectious diseases), electrocardiograms, and definitive histopathological or cytological diagnoses. The primary exclusion criteria included any abnormal findings in medical evaluations, a documented history of significant diseases, or a history of severe drug allergies. Participants meeting the inclusion criteria were subjected to screening, while those who conformed to any of the exclusion criteria were excluded from the study.

### Study drugs

The test formulation (specifications: each capsule contained 25 mg FT, 7.25 mg CDHP, and 24.5 mg OXO; batch number: 16L0013E29) was developed by Qilu Pharmaceutical Co., Ltd. The reference formulation (specifications: each capsule contained 25 mg FT, 7.25 mg CDHP, and 24.5 mg OXO; batch number: 6K97B) was developed by Taiho Pharmaceutical Co., Ltd. Both drugs, provided by Qilu Pharmaceutical Co., Ltd., were from commercially available batches with valid certificates of analysis and were stored in a sealed container at a controlled room temperature of 15°C–25°C until further use. The inactive ingredients in both the test and reference formulations were identical, consisting of lactose monohydrate and magnesium stearate. The dose administered to participants was expressed as the FT content.

### Blood sampling and medical supervision

Blood samples for PK evaluation were collected in vacuum anticoagulant tubes at 0 h (within 60 min before dosing) and 0.5, 1, 1.5, 2, 2.5, 3, 4, 6, 8, 10, 12, 24, and 48 h post-dosing. At each specified time point, 3 mL of blood sample was drawn. Fourteen blood samples, amounting to a total volume of 42 mL, were collected per cycle. The samples underwent centrifugation at 1700 g (maintained at a temperature range of 2°C–8°C) for 10 min to facilitate plasma separation. Subsequently, the samples were promptly stored at −70°C (not exceeding −60°C) within 120 min post-centrifugation.

Clinical researchers diligently monitored adverse events (AEs) at each participating center. Safety assessments were executed comprehensively, incorporating physical examinations, monitoring vital signs and electrocardiogram (ECG) recordings, AE reporting, analysis of clinical laboratory parameters, and documenting concurrent medication usage. AEs were meticulously classified according to version 4.03 of the Common Terminology Criteria for Adverse Events (CTCAE) and subjected to analysis using descriptive statistical methods. All AEs manifesting after medication administration were rigorously documented. Comprehensive follow-up evaluations were systematically conducted to assess the timing of onset, severity, duration, interventions implemented, and outcomes, continuing until the resolution of symptoms, disease stabilization, or loss of follow-up. Safety assessments were conducted for participants who withdrew from the study early or within 3 days after completing the second round of blood collection. These assessments included monitoring of vital signs, physical examination, laboratory tests, and 12-lead electrocardiograms, all of which were integral components in evaluating the overall safety of the study.

### Chromatographic conditions

The determination of FT, CDHP, and 5-FU in blood was performed under the following conditions: the mobile phase consisted of 100% water containing 0.005% formic acid (mobile phase A) and a mixture of methanol and acetonitrile (2:1, v/v) (mobile phase B). The flow rate was set to 0.8 mL/min, and the gradient was programmed as follows: 5% mobile B for 3.1 min; 40% mobile B for 0.3 min, and then returned to 5% mobile phase B, which was maintained for 2.4 min. Chromatographic separation was achieved using an Eclipse XDB Phenyl column (5 μm, 4.6 mm × 150 mm), which provided satisfactory separation efficiency. The autosampler and column temperatures were set at 4°C and 40°C, respectively, with an injection volume of 10 μL.

OXO was determined in blood, which was performed under the following conditions: the aqueous solution containing 5 mM ammonium acetate (mobile phase A) and acetonitrile (mobile phase B). The flow rate was set to 0.8 mL/min, and the gradient was programmed as follows: 30% mobile B for 2.8 min, 60% mobile B for 0.01 min, then returned to 30% mobile phase B, and maintained for 0.6 min. Chromatographic separation was achieved using XDB-C18 (1.8 μm, 4.6 mm × 50 mm), which provided satisfactory separation efficiency. The autosampler and column temperatures were set at 4°C and 35°C, respectively, with an injection volume of 10 μL.

### Mass spectrometric conditions

The determination of FT, CDHP, and 5-FU in blood was conducted utilizing multi-reaction monitoring in the negative ion mode, employing an electric spray ion source. Optimized parameters included an ion spray voltage of 4500 V, GS1 and GS2 pressures set at 60 psi each, a curtain gas pressure of 35 psi, a collision gas pressure of 8 psi, and a source temperature of 35°C. Multiple reaction monitoring transitions were as follows: for FT and FT-13C,15N2, m/z 199.1–42.1 and m/z 202.0–44.0, respectively; for 5-FU and 5-FU-13C,15N2, m/z 128.8–42.1 and m/z 132.0–44.0, respectively; and CDHP and CDHP-13C3, m/z 143.9–64.0 and m/z 147.0–67.0, respectively.

The determination of OXO in blood was performed in the positive ion mode using an atmospheric pressure chemical ionization source. Optimized parameters included a declustering voltage of 80 V, GS1 set to 65 psi, curtain gas pressure of 35 psi, collision gas pressure of 10 psi, and an ion source temperature of 600°C. Multiple reaction monitoring transitions for OXO and OXO-13C2,15N3 were m/z 490.0–259 and m/z 495–262, respectively.

### Determination of FT, CDHP, 5-FU, and OXO

FT, CDHP, 5-FU, and OXO plasma concentrations were quantified using a validated liquid chromatography–mass spectrometry (LC-MS/MS) method. The method exhibited a linear range of 10.0–3,000 ng/mL for FT, with a lower limit of quantification (LLOQ) of 10.0 ng/mL. Precision, expressed as the coefficient of variation (% CV), ranged from 0.8% to 4.5% for each concentration quality control (QC) sample, while the mean bias from theoretical concentrations ranged between −7.7% and 4.7% for FT.

CDHP’s linear range was 2.0–600 ng/mL, with an LLOQ value of 2.0 ng/mL. Precision ranged from 0.9% to 4.2% (% CV) for each QC sample, and the mean bias ranged between −6.3% and 4.6%.

Similarly, 5-FU exhibited a linear range of 1.0–300 ng/mL, with an LLOQ value of 1.0 ng/mL. Precision ranged from 0.8% to 4.2% (% CV) for QC samples, and the mean bias ranged between −7.2% and 5.5%.

For OXO, the linear range was 2.0–200 ng/mL, with an LLOQ value of 2.0 ng/mL. The precision ranged from 0.3% to 9.9% (% CV) for the QC samples, and the mean bias ranged between −13.4% and 8.4%. These results demonstrate the robustness and reliability of the LC-MS/MS method for the quantification of these analytes in plasma.

### PK and BE analysis

This clinical study is a BE study with PK parameters as the endpoint. The PK parameters assessed included AUC_0–t_, AUC_0– ∞ _, C_max_, the elimination half-life (t_1/2_), and the time to achieve C_max_ (T_max_). Based on blood concentration measurements, the PK parameters for FT, CDHP, 5-FU, and OXO were calculated using WinNonlin version 6.4 through a non-compartmental analysis approach. The area under the curve (AUC) was determined by employing the linear trapezoidal interpolation method. The experimental results were primarily analyzed using descriptive statistical methods with SAS software (SAS Institute, version 9.4). A P-value of <0.05 was considered to indicate statistical significance.

Biological equivalence was rigorously assessed using a double one-sided t-test and a confidence interval methodology. The PK parameters AUC_0-t_, AUC_0- ∞ _, and C_max_ obtained from subjects at different cycles were log-transformed and analyzed by ANOVA. Ninety percent confidence intervals (CIs) were calculated for the geometric mean ratio of the test formulation to the geometric mean of the reference formulation. According to the guidance principles of the US Food and Drug Administration (FDA), biological equivalence is established if the 90% CIs for the geometric mean ratios of AUC_0-t_, AUC_0- ∞ _, and C_max_ of the test formulation to the reference formulation fall within the range of 80%–125% (FDA, 2014). If either end of the range is outside this interval, no equivalence can be concluded.

## Results

### Subjects’ demographic characteristics

**FIGURE 1:**
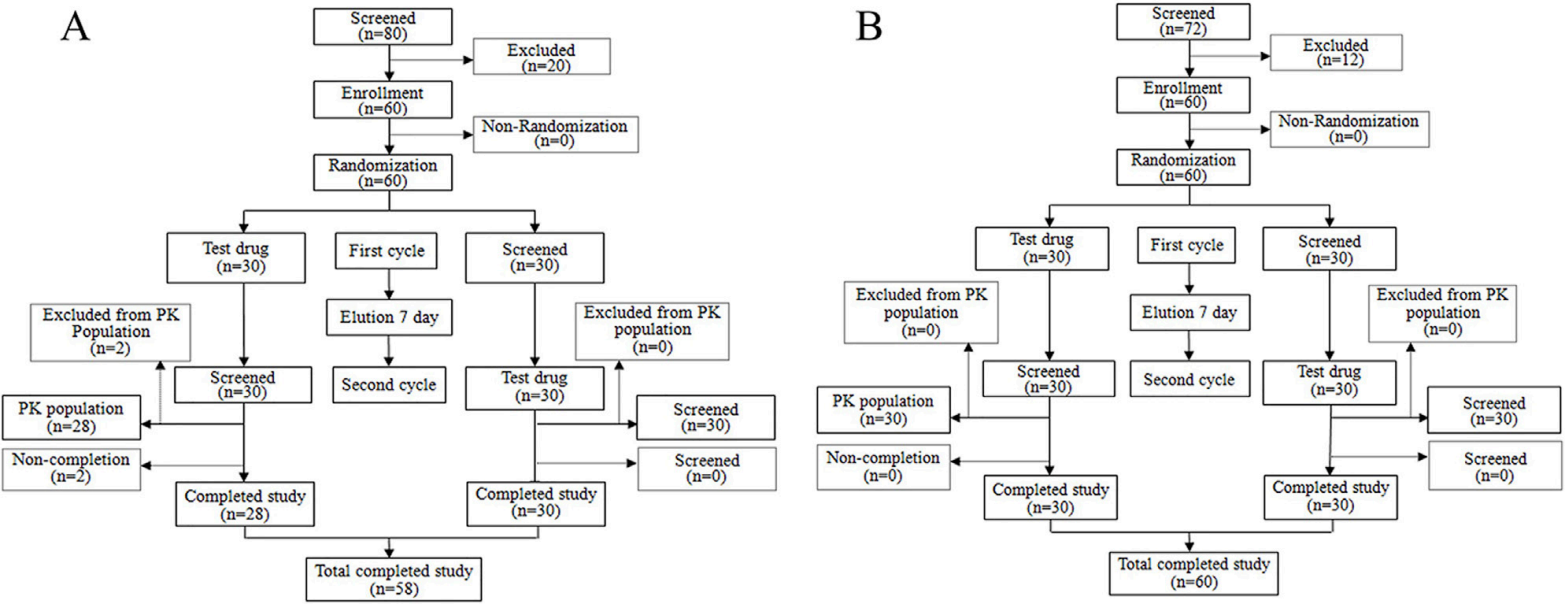
Study design and study flow diagram. Study design and study flow diagram in the fasting condition **(A)** and study design and study flow diagram in the fed condition **(B)**.

A total of 80 volunteers were screened for the fasting trial. Subsequently, 20 subjects were excluded based on the predefined inclusion and exclusion criteria, culminating in the final inclusion and randomization of 60 subjects. For the fed trial, 72 volunteers underwent initial screening, from which 12 subjects were excluded, adhering to the specified inclusion and exclusion criteria, thereby resulting in the eventual inclusion and randomization of 60 subjects. Throughout the study period, two subjects withdrew from the fasting trial for personal reasons before receiving a single administration. A comprehensive flowchart illustrating the distribution of subjects is depicted in Figure 1, while Table 1 elaborately delineates the demographic characteristics of each group.

**TABLE 1:** Demographic characteristics for each group.

Parameter	Fasting	Fed
N	60	60
Gender, n (%)		
Male subjects	37 (61.7)	22 (36.7)
Female subjects	23 (38.3)	38 (63.3)
Age (years)		
Mean ± SD	53.9 ± 9.26	55.1 ± 8.78
Min–Max	29–69	33–72
Height (cm)		
Mean ± SD	165.33 ± 6.015	163.78 ± 6.303
Min–Max	150.0–181.0	150.0–180.0
Weight (kg)		
Mean ± SD	63.11 ± 7.445	63.16 ± 6.651
Min–Max	50.7–78.8	52.0–83.0
BMI (kg/m^2^)		
Mean ± SD	23.07 ± 2.331	23.56 ± 2.228
Min–Max	18.3–27.5	19.1–27
BSA (m^2^)		
Mean ± SD	1.663 ± 0.1187	1.655 ± 0.1094
Min–Max	1.5009–1.9476	1.5015–1.9801

N, number of subjects; SD, standard deviation; Min, minimum; Max, maximum; BMI, body mass index; BSA, body surface area.

### PK parameters and plasma concentration–time curve

In this study, two participants in the fasting cohort elected to terminate their involvement before initiating medication administration. Consequently, their data were omitted from the PK concentration set (PKCS) and the PK parameter set (PKPS). The residual cohort, comprising 118 participants, completed the trial, warranting their inclusion in subsequent analyses of the PKCS and PKPS. Following the oral administration of S-1, the gastrointestinal tract rapidly absorbed both FT and CDHP. Under fasting conditions, T_max_ was recorded to occur within an approximate 0.5–1 h post-dosing range.

**FIGURE 2:**
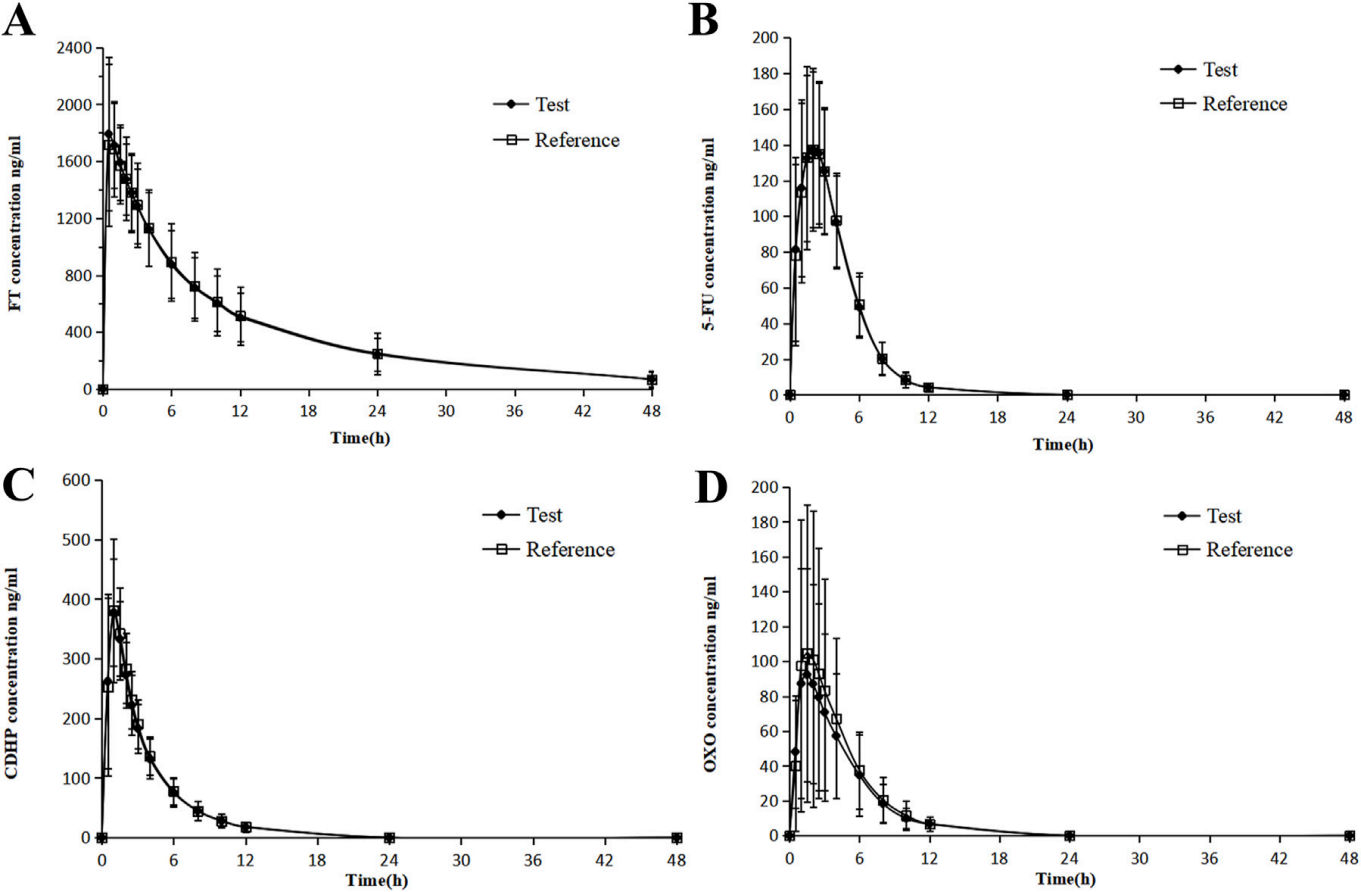
Mean blood concentration (±SD) time curve for FT **(A)**, 5-FU **(B)**, CDHP **(C)**, OXO, and **(D)** after oral single-dose administration of S-1 of the test and reference formulations during fasting.

**FIGURE 3:**
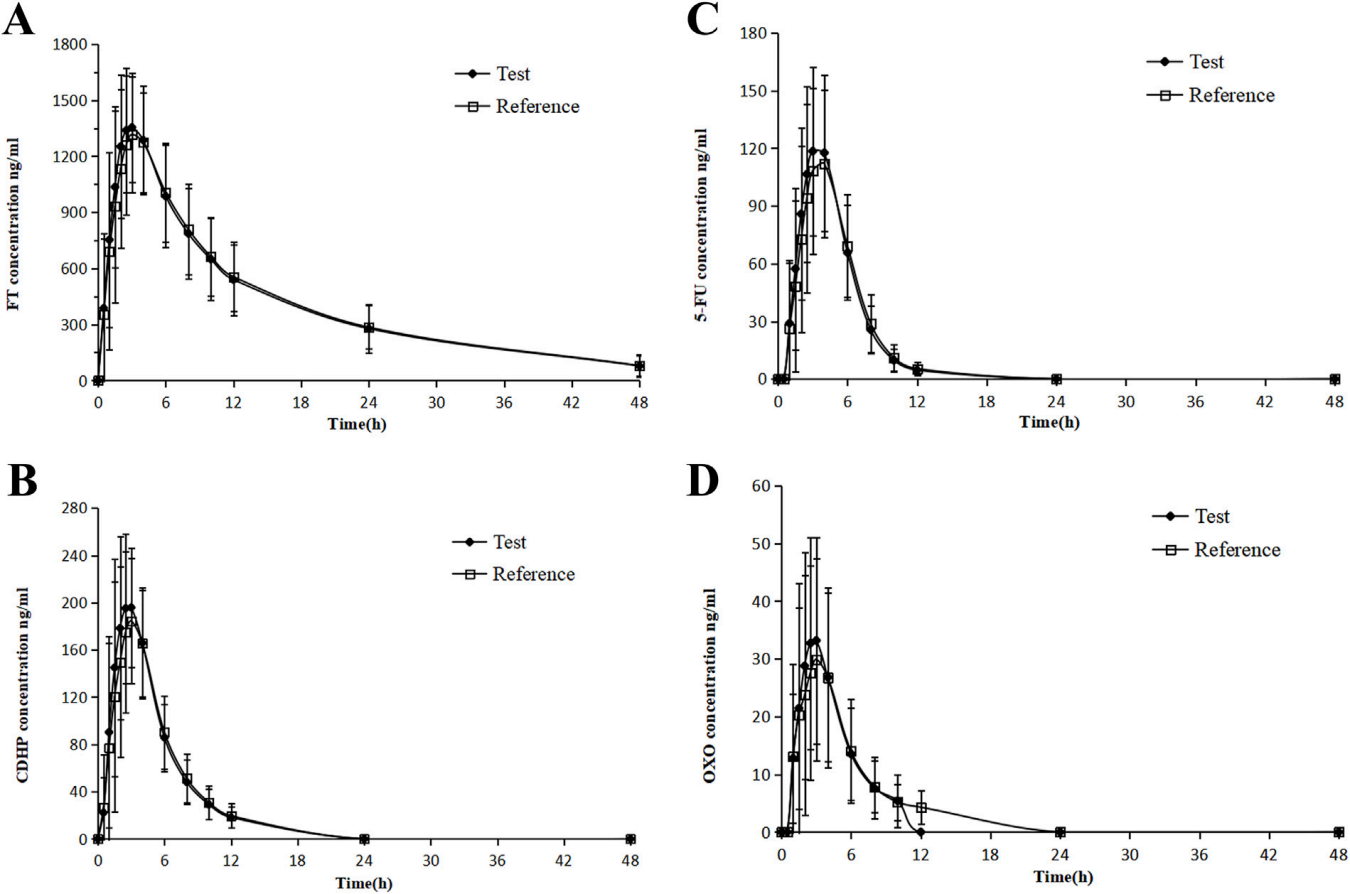
Mean blood concentration (±SD) time curve for FT **(A)**, 5-FU **(B)**, CDHP **(C)**, OXO, and **(D)** after oral single-dose administration of S-1 of the test and reference formulations during the fed state.

In contrast, T_max_ for FT and CDHP was delayed to approximately 3 h, following the consumption of a high-fat meal, with similar extensions observed for the T_max_ of 5-FU and OXO, a variation ascribed to the impact of dietary intake. Following the consumption of a high-fat meal, the area under the concentration–time curve (AUC) for FT remained largely stable, with a slight decrease observed in the AUC values for 5-FU and CDHP, accompanied by a significant reduction in the AUC for OXO. Table 2 outlines the critical PK parameters of FT, CDHP, 5-FU, and OXO in both fasting and fed states. Figures 2, 3 depict the average plasma concentration–time curves of FT, CDHP, 5-FU, and OXO obtained after a single oral trial and referenced S-1 in the fasting and fed groups.

**TABLE 2:** Summary of pharmacokinetic parameters of FT, 5-FU, CDHP, and OXO after a single dose of the test drug or reference drug in the fasting and fed states.

	Parameter	Fasting (N = 58)		Postprandial (N = 60)	
		Test	Reference	Test	Reference
FT	C_max_ (ng·mL^−1^)	1,937.931 ± 370.603	1,936.207 ± 353.528	1,483.317 ± 307.594	1,458.450 ± 302.144
	T_max_ (h)*	0.504 (0.476, 2.002)	0.506 (0.476, 2.477)	2.976 (0.489, 6.000)	2.988 (0.486, 6.001)
	t_1/2_ (h)	11.477 ± 2.665	11.339 ± 2.583	11.899 ± 2.932	11.977 ± 2.878
	AUC_0-t_ (h·ng·mL^−1^)	19,591.485 ± 6,308.448	19,918.399 ± 7,336.727	19,820.580 ± 6,660.536	19,946.691 ± 6,337.422
	AUC_0- ∞ _ (h·ng·mL^−1^)	20,880.758 ± 7,434.940	21,219.131 ± 8,789.984	21,376.093 ± 8,022.792	21,517.858 ± 7,544.771
5-FU	C_max_ (ng·mL^−1^)	144.516 ± 46.353	143.741 ± 46.021	130.488 ± 44.060	124.642 ± 42.595
	T_max_ (h)*	2.001 (0.479, 3.002)	1.988 (0.500, 4.003)	3.477 (1.500, 6.002)	3.963 (1.481, 6.001)
	t_1/2_ (h)	1.674 ± 0.233	1.676 ± 0.264	1.523 ± 0.277	1.564 ± 0.376
	AUC_0-t_ (h·ng·mL^−1^)	698.366 ± 183.996	701.208 ± 189.871	613.609 ± 169.554	594.726 ± 162.900
	AUC_0- ∞ _ (h·ng·mL^−1^)	709.029 ± 184.447	712.128 ± 189.904	624.440 ± 169.699	608.061 ± 163.428
CDHP	C_max_ (ng·mL^−1^)	405.138 ± 79.773	417.034 ± 90.058	230.350 ± 60.191	223.248 ± 64.576
	T_max_ (h)*	1.000 (0.476, 2.002)	1.000 (0.485, 2.477)	2.507 (0.978, 6.000)	2.984 (0.979, 6.001)
	t_1/2_ (h)	3.246 ± 0.925	3.144 ± 0.715	3.378 ± 0.994	3.500 ± 1.748
	AUC_0-t_ (h·ng·mL^−1^)	1,448.510 ± 296.449	1,481.437 ± 295.328	1,137.733 ± 296.868	1,120.538 ± 292.284
	AUC_0- ∞ _ (h·ng·mL^−1^)	1,486.099 ± 295.582	1,518.060 ± 293.937	1,168.818 ± 294.950	1,150.551 ± 289.607
OXO	C_max_ (ng·mL^−1^)	105.086 ± 70.299	121.717 ± 94.101	37.370 ± 20.256	36.162 ± 22.916
	T_max_ (h)*	1.493 (0.479, 6.001)	1.979 (0.978, 7.951)	2.978 (1.479, 6.000)	2.996 (1.000, 6.009)
	t_1/2_ (h)	3.043 ± 3.335	2.449 ± 0.887	3.578 ± 2.682	4.904 ± 8.233
	AUC_0-t_ (h·ng·mL^−1^)	474.954 ± 259.736	528.310 ± 333.224	175.203 ± 97.561	165.382 ± 89.518
	AUC_0- ∞ _ (h·ng·mL^−1^)	497.303 ± 261.111	553.429 ± 337.647	201.364 ± 111.341	195.680 ± 93.603

N, number of subjects; *C*_max_, maximum observed drug concentration in the plasma; T_max_, time from administration to the maximum observed concentration of the analyte in the plasma; t_1/2_, terminal half-life of the analyte in the plasma; AUC_0-t_, AUC of the analyte in the plasma over the time interval from time zero to the last measurable concentration; AUC_0- ∞ _, area under the curve from 0 to infinity.

* Indicates that the data is expressed as median (minimum, maximum).

### BE results

As outlined in Tables 3, 4, with the exception of OXO under fasting conditions, where the least squares mean ratios for C_max_, AUC_0-t_, and AUC_0- ∞_ ranged from 90.35% to 93.88%, all other ratios for these PK parameters were close to 100%. Under both fasting and fed states, the 90% CIs for C_max_, AUC_0-t_, and AUC_0- ∞_ of FT, 5-FU, CDHP, and OXO fell within the established BE range of 80%–125%. These results substantiate the BE of the S-1 test formulation relative to the reference formulation under both fasting and fed conditions.

**TABLE 3:** Results of the equivalence determination of the test drug and reference drug during fasting.

	Parameter	GLS mean			Intra-CV (%)	90% CI of ratio (%)
		Test (N = 58)	Reference (N = 58)	T/R (%)		
FT	C_max_ (ng·mL^−1^)	1,687.35	1,677.62	100.58	6.8	95.86,105.53
	AUC_0-t_ (h·ng·mL^−1^)	20,252.07	20,046.37	101.03	4.9	97.61,104.56
	AUC_0- ∞ _(h·ng·mL^−1^	21,457.87	21,288.91	100.79	5.5	96.96,104.78
5-FU	C_max_ (ng·mL^−1^)	136.13	135.01	100.82	10.0	97.61,104.14
	AUC_0-t_ (h·ng·mL^−1^)	673.87	669.14	100.71	8.6	97.94,103.55
	AUC_0- ∞ _(h·ng·mL^−1^	684.94	680.63	100.63	8.4	97.91,103.43
CDHP	C_max_ (ng·mL^−1^)	386.98	398.22	97.18	14.4	92.18,102.45
	AUC_0-t_ (h·ng·mL^−1^)	1,421.89	1,466.94	96.93	7.3	94.36,99.57
	AUC_0- ∞ _(h·ng·mL^−1^	1,459.52	1,503.64	97.07	7.2	94.52,99.68
OXO	C_max_ (ng·mL^−1^)	90.33	99.98	90.35	38.3	80.10,101.91
	AUC_0-t_ (h·ng·mL^−1^)	424.60	455.07	93.31	35.3	83.45,104.32
	AUC_0- ∞ _(h·ng·mL^−1^)	446.96	476.08	93.88	34.1	84.26,104.60

N, number of subjects; GLS mean, geometric least squares mean; CI, confidence interval; AUC_0-t_, AUC of the analyte in the plasma over the time interval from time zero to the last measurable concentration; AUC_0- ∞ _, area under the curve from 0 to infinity; C_max_, maximum observed drug concentration in the plasma; CV%, within-subject coefficient of variation.

Test S-1: (manufactured by Qilu Pharmaceutical Co., Ltd., Shandong, China). Reference S-1: TS-1 (Taiho Pharmaceutical Co., Ltd., Tokushima Plant, Japan).

**TABLE 4:** Results of the equivalence determination of the test drug and reference drug during feeding.

	Parameter	GLS mean			Intra-CV (%)	90% CI of ratio (%)
		Test (n = 60)	Reference (n = 60)	T/R (%)		
FT	C_max_ (ng·mL^−1^)	1,448.79	1,425.87	101.61	8.2	99.04,104.24
	AUC_0-t_ (h·ng·mL^−1^)	18,812.13	19,116.26	98.41	7.3	96.20,100.67
	AUC_0- ∞ _(h·ng·mL^−1^	20,067.18	20,442.42	98.16	7.3	95.96,100.42
5-FU	C_max_ (ng·mL^−1^)	123.01	117.55	104.64	14.5	100.14,109.34
	AUC_0-t_ (h·ng·mL^−1^)	590.79	571.35	103.40	11.2	99.95,106.97
	AUC_0- ∞ _(h·ng·mL^−1^	602.08	585.06	102.91	10.7	99.60,106.32
CDHP	C_max_ (ng·mL^−1^)	222.66	214.14	103.98	19.6	98.00,110.32
	AUC_0-t_ (h·ng·mL^−1^)	1,103.93	1,084.00	101.84	10.7	98.58,105.21
	AUC_0- ∞ _(h·ng·mL^−1^	1,136.02	1,115.79	101.81	10.3	98.68,105.04
OXO	C_max_ (ng·mL^−1^)	32.05	30.24	105.97	31.9	96.38,116.52
	AUC_0-t_ (h·ng·mL^−1^)	149.94	141.25	106.15	28.7	97.43,115.65
	AUC_0- ∞ _(h·ng·mL^−1^	176.58	174.56	101.16	27.0	93.30,109.68

N, number of subjects; GLS mean, geometric least squares mean; CI, confidence interval; AUC_0-t_, AUC of the analyte in the plasma over the time interval from time zero to the last measurable concentration; AUC_0- ∞ _, area under the curve from 0 to infinity; C_max_, maximum observed drug concentration in the plasma; CV%, within-subject coefficient of variation.

Test S-1: (manufactured by Qilu Pharmaceutical Co., Ltd., Shandong, China). Reference S-1: TS-1 (Taiho Pharmaceutical Co., Ltd., Tokushima Plant, Japan).

### Safety and tolerability

In the fasting trial, a total of 43 AEs (22 caused by the test formulation and 21 caused by the reference formulation) occurred in 25 of the 58 subjects who entered the safety analysis set, of which 12 AEs (five caused by the test formulation and seven caused by the reference formulation) were judged to be drug-related. The most common drug-related AEs observed throughout the study were decreased WBC, increased AST, increased blood urea, decreased neutrophil count, increased ALT, and anemia. There were no serious adverse events (SAEs) or drug-related deaths and no AEs, leading to discontinuation. Detailed AEs are shown in Table 4.

In the fed trial, a total of 24 AEs (17 caused by the test formulation and 7 caused by the reference formulation) occurred in 16 of the 60 subjects who entered the safety analysis set, of which 14 AEs (nine caused by the test formulation and five caused by the reference formulation) were judged to be drug-related. The most common drug-related AEs observed throughout the study were decreased WBC, increased AST, decreased neutrophil count, headache, increased blood glucose, increased ALT, and anemia. There were no SAEs or drug-related deaths and no AEs leading to discontinuation. Detailed AEs are shown in Tables 5, 6.

**TABLE 5:** Summary of drug-related AEs in the fasting group.

Adverse reaction	Test drug (N = 58)		Reference drug (N = 58)	
	Number of subjects (%)	N	Number of subjects (%)	N
Total AEs	22 (37.9)	22	19 (32.8)	21
AEs related to drugs	6	6	10	10
WBC count decreased	3 (5.2)	3	5 (8.6)	5
Neutrophil count decreased	1 (1.7)	1	2 (3.4)	2
Blood urea increased	1 (1.7)	1	1 (1.7)	1
AST increased	1 (1.7)	1	0	0
ALT increased	0	0	1 (1.7)	1
Anemia	0	0	1 (1.7)	1
AEs not related to drugs	16	16	9	11
Hypertension	2 (3.4)	2	2 (3.4)	4
Blood pressure increased	2 (3.4)	2	1 (1.7)	1
Cough	1 (1.7)	1	1 (1.7)	1
Upper respiratory tract infection	0	0	2 (3.4)	2
Decreased appetite	1 (1.7)	1	0	0
WBC count increased	1 (1.7)	1	0	0
Hematuria	1 (1.7)	1	0	0
Weight reduction	1 (1.7)	1	0	0
Neutrophil percentage increased	1 (1.7)	1	0	0
Neutrophil count increased	1 (1.7)	1	0	0
Lymphedema	1 (1.7)	1	0	0
Nausea	1 (1.7)	1	0	0
Vomit	1 (1.7)	1	0	0
Atrial fibrillation	1 (1.7)	1	0	0
Sinus tachycardia	1 (1.7)	1	0	0
Fall	0	0	1 (1.7)	1
Eye contusion	0	0	1 (1.7)	1
ECG ST-T wave changes	0	0	1 (1.7)	1
At least grade three adverse reactions	2 (3.4)	2	1 (1.7)	1
SAE	0	0	0	0
Drug-related death	0	0	0	0

AEs, adverse events; SAEs, serious adverse events; N, number of adverse events.

**TABLE 6:** Summary of drug-related AEs in the fed group.

Adverse reaction	Test drug (N = 60)		Reference drug (N = 60)	
	Number of subjects (%)	N	Number of subjects (%)	N
Total AEs	21 (35.0)	22	8 (13.3)	9
AEs related to drugs	12 (20.0)	12	5 (8.3)	6
WBC count decreased	2 (3.3)	2	1 (1.7)	2
AST increased	2 (3.3)	2	1 (1.7)	1
Blood glucose increased	2 (3.3)	2	1 (1.7)	1
Neutrophil count decreased	2 (3.3)	2	1 (1.7)	1
Anemia	1 (1.7)	1	1 (1.7)	1
ALT increased	1 (1.7)	1	0	0
Headaches	1 (1.7)	1	0	0
Neutrophil count decreased	1 (1.7)	1	0	0
AEs not related to drugs	9 (15.0)	10	3 (5.0)	3
Diseases of the blood and lymphatic system	2 (3.3)	2	1 (1.7)	1
Respiratory, thoracic, and mediastinal diseases	1 (1.7)	2	0	0
Cholecystitis	1 (1.7)	1	0	0
Blood alkaline phosphatase increased	1 (1.7)	1	0	0
Heart disease	1 (1.7)	1	0	0
Sinus bradycardia	1 (1.7)	1	0	0
Catarrhal inflammation	1 (1.7)	1	0	0
Hemoptysis	1 (1.7)	1	0	0
Gastrointestinal system disorders	0	0	1 (1.7)	1
Constipation	0	0	1 (1.7)	1
At least grade three adverse reactions	1 (1.7)	1	1 (1.7)	1
SAE	0	0	0	0
Drug-related death	0	0	0	0

AEs, adverse events; SAE, serious adverse events; N, number of adverse events.

The types of AEs were similar between the two formulations under fasting and fed conditions. After the end of the study, all AEs returned to normal or remained stable. The research results showed that both formulations are safe.

## Discussion

S-1 is a composite drug comprising CDHP, OXO, and FT metabolically converted to 5-FU *in vivo*. Consequently, BE studies focusing on S-1 necessitate the evaluation of PK parameters for FT, CDHP, OXO, and 5-FU. Notably, the t_1/2_ of FT in prior PK analyses was approximately 13.1 ± 3.1 h in the test substance; therefore, in the current investigation, blood samples were collected up to 48 h post-administration, exceeding the minimum threshold of three times the t_1/2_ endpoint for FT. Moreover, the mean AUC_0-t_/AUC_0- ∞_ ratios for FT, 5-FU, CDHP, and OXO in our study were consistently between 92.9% and 95.8%, confirming that the duration of sample collection ensures an accurate assessment of drug exposure. Importantly, none of the compounds FT, CDHP, OXO, and 5-FU were detectable in plasma samples prior to administration in the second cycle of the clinical trial, indicating that the 7-day washout period, based on t_1/2_ values from earlier PK studies, was adequate to achieve complete clearance of the investigational drugs from the bloodstream, following the initial treatment cycle.

S-1’s primary anticancer ingredient is the prodrug FT, which converts to its active form 5-FU through hydroxylation. In our study, formulations were administered, where each capsule contained 25 mg of FT, 7.25 mg of CDHP, and 24.5 mg of OXO, with subjects ingesting two capsules per dose. Given that the participants’ average body surface area (BSA) was 1.66 m^2^, the BSA-normalized dose of tegafur employed in our research was established to be 30.12 mg/m^2^. Upon standardizing the dose to 40 mg/m^2^, we observed that under fasting conditions, the C_max_ and AUC_0-t_ values for FT were notably higher, ranging from 2571.32 to 2573.61 ng/mL and 26,017.91 to 26,452.06 h·ng/mL, respectively, exceeding those reported by Lee et al. (2019). Under fed conditions, the corresponding values were 1936.85–1969.88 ng/mL for C_max_ and 26,322.15–26,489.63 h·ng/mL for AUC_0-t_, consistent with the findings reported by Chen et al. (2020).

Reports indicate that FT exposure is comparable in East Asians and is reduced in Europeans (Chuah et al., 2011). Compared to European and American patients under the fed condition (Peters et al., 2003; Hoff et al., 2003), Chinese individuals exhibit elevated AUC_0-t_ levels of FT, a trend mirrored by Japanese patients (Zhuang et al., 2013). This disparity is likely attributable to the conversion of FT to 5-FU *in vivo*, predominantly via the hydroxylation activity of CYP2A6—a highly polymorphic enzyme that exhibits common allelic variants (CYP2A6*4, *7, and *9) at increased frequencies in East Asians (Ikeda et al., 2000; Yoshida et al., 2003). These variants are linked to decreased enzymatic activity, which may contribute to reduced FT activation, thereby explaining the reduced 5-FU exposure observed in Asians during phase I trials. Although variations in CYP2A6 influence FT pharmacokinetics, they do not significantly affect the pharmacokinetics of 5-FU (Fujita et al., 2008; Kim et al., 2011). A real-world study demonstrated a significant correlation between the AUC of 5-FU and CDHP concentrations but not with the CYP2A6 genotype, suggesting that 5-FU exposure is primarily governed by CDHP levels rather than by the biotransformation of FT (Fujita and Sasaki, 2014; Hirose et al., 2010).

5-FU is a pyrimidine analog antimetabolite drug that disrupts nucleoside metabolism and integrates into RNA and DNA and is a commonly used antitumor drug in clinical practice (Sun et al., 2021; Longley et al., 2003). Following parenteral administration of 5-FU, the drug is rapidly distributed and swiftly eliminated, with a half-life of approximately 8–20 min (Diasio and Harris, 1989). The clearance of 5-FU from a patient’s system is influenced by the rate at which FT is converted to 5-FU and the level of inhibition imposed on dihydropyrimidine dehydrogenase by CDHP (Zhuang et al., 2013). This study demonstrated that the half-life of 5-FU was significantly prolonged, following a single dose of two tablets of S-1. These findings suggest that S-1 plays an active role in extending the duration of action of 5-FU in the body, highlighting its potential therapeutic implications.

CDHP, a potent inhibitor of DPD, primarily functions to impede the degradation of 5-FU, thereby prolonging its pharmacological activity. In our study, we meticulously examined the impact of food intake on the PK parameters of CDHP. It was observed that the presence of food significantly reduced C_max_, a phenomenon likely attributable to the modulation of drug absorption rates and extent by food. Concurrently, T_max_ was notably prolonged, which may be associated with delayed gastric emptying, alterations in intestinal pH, or modifications in the activity of intestinal drug transport proteins induced by food intake (Deng et al., 2017). However, despite the pronounced effects on C_max_ and T_max_, the influence of food on the half-life and total exposure to CDHP was comparatively minimal. This suggests that once absorbed, the metabolic clearance of CDHP *in vivo* is minimally affected by food. These findings hold significant implications for clinical application, indicating that physicians may need to adjust the timing or dosage of CDHP administration based on dietary conditions to maximize therapeutic efficacy. Additionally, given that CDHP is predominantly metabolized by the kidneys and excreted via glomerular filtration into the urine, a preclinical assessment of renal function is imperative to mitigate the risk of impaired renal function (Fujita et al., 2009). Impaired renal function may elevate CDHP concentrations, potentially leading to increased 5-FU levels and unpredictable toxicities. Dosage adjustments should be considered necessary.

OXO blocks an enzyme called orotate phosphoribosyl transferase and is highly concentrated in the gastrointestinal (GI) tract. This helps OXO reduce GI problems caused by the drug 5-FU (Koizumi et al., 2010). In our study, we analyzed the pharmacological properties of OXO in comparison with three other compounds. OXO exhibited the greatest variability, with the within-subject CV% for both C_max_ and AUC exceeding 30%. This finding is consistent with other PK studies of S-1, where similar levels of variability have been observed (Chen et al., 2020; Lee et al., 2019). Consequently, during the sample size calculation for this study, the variability of OXO was the primary factor considered.

Additionally, our study specifically addresses the impact of dietary intake on OXO’s PK parameters. Data indicate that postprandial administration of a high-fat meal significantly reduces the C_max_ and AUC of OXO, which is generally consistent with the US Max E study (Scheulen et al., 2012). These results suggest that food intake, particularly high-fat food, may substantially influence the absorption and metabolism of OXO, potentially through alterations in the gastrointestinal environment, interference with drug solubility, or disruption of the intestinal drug transport system. Therefore, we recommend administering S-1 on an empty stomach to enhance the bioavailability of OXO and reduce the gastrointestinal side effects associated with 5-FU. Additionally, a European study indicated that the C_max_ and AUC_0-t_ values for OXO, following fasting administration of S-1, were lower than those observed in our study (Hoff et al., 2003), which may explain the higher incidence of gastrointestinal side effects observed in European populations when taking S-1.

Understanding the characteristics of drugs is crucial for interpreting their PK profiles in the body. The drug FT exhibits alkalinity and has a LogP value of −0.48 (Lee et al., 1994). Although drugs with a LogP value of less than 0 are typically classified as hydrophilic, FT demonstrates significant lipophilicity (Lee et al., 1994). This phenomenon may be attributed to FT being a derivative of 5-FU, with structural modifications that enhance its interaction with lipid components in biological membranes. Additionally, the metabolic conversion of FT to 5-FU *in vivo* confers certain lipophilic properties to FT. Lipophilic drugs tend to accumulate in adipose tissue, which increases the distribution volume of FT and consequently prolongs its t_1/2_. On the other hand, the molecular structure of CDHP contains a hydroxyl group (−OH) and a chlorine atom (−Cl), which impart a weakly acidic characteristic. Weakly acidic drugs are less likely to ionize in the gastric environment, thus favoring their existence in a non-ionized form. This characteristic facilitates the absorption of drugs through cell membranes, providing a plausible explanation for the rapid absorption of CDHP under fasting conditions, as observed in our study with a T_max_ value of approximately 1 h. OXO, a salt, exhibits acidity upon dissolution in water. In this study, we found that food intake significantly reduced the AUC of OXO. The likely cause is that food intake increases the gastric pH (Sekine et al., 2021), leading to increased ionization of the acidic drug. Although solubility is enhanced, the proportion of the non-ionized form decreases, which may reduce transmembrane absorption and consequently lower drug exposure.

Clinical investigations revealed that the gastrointestinal toxicity associated with S-1 is notably mild. In a comprehensive Phase III trial conducted across European and American cohorts (Koizumi et al., 2008), adverse gastrointestinal events—namely diarrhea, stomatitis, and vomiting of grade 3 severity or higher—were reported in less than 5% of cases when S-1 was administered on an empty stomach in conjunction with cisplatin. These findings substantiate the protective role of OXO in the gastrointestinal tract. The main adverse reactions in this study include decreased WBC, increased AST, increased blood urea, decreased neutrophil count, and increased ALT, headache, and anemia. It is noteworthy that S-1 was administered as a single dose in this study, and the safety assessment was thus confined to this single administration. However, gastrointestinal toxicity is typically associated with the prolonged use of S-1, suggesting that long-term treatment may require additional monitoring and management strategies beyond the scope of the present investigation. Notably, while hematological toxicity emerged as the predominant adverse effect in Japanese patients, gastrointestinal disturbances, particularly diarrhea, were more pronounced in Western cohorts (Haller et al., 2008). Another comparison of uracil ⁄ FT and leucovorin in metastatic colorectal cancer between Japanese and North American patients showed that both ethnic groups had comparable 5-FU exposure when compared according to the body surface area. However, American patients had a higher incidence of diarrhea (Shirao et al., 2004). Additionally, a significant correlation was identified between prolonged use of the S-1 antineoplastic drug combination and ocular complications. Elevated levels of FT in tears and increased tear volume have been linked to symptoms such as excessive tearing. Proactive communication about these potential ocular side effects to patients and enhanced information sharing among healthcare providers are recommended to mitigate the escalation of such issues (Kuriki et al., 2019).

This study meticulously investigated the PK properties of the newly developed S-1 formulation. It assessed its BE with the established brand of S-1 in a large cohort of 118 cancer patients under fasting and postprandial conditions. The findings demonstrate that all components of the new formulation achieved BE in both conditions, with notable modulation by food intake on the PK of CDHP and OXO. Furthermore, both formulations were characterized by their favorable safety profiles and tolerability.

Although our study provides valuable insights into the PK and BE of the S-1 formulation, it is essential to consider the broader implications for clinical practice and global applications. In terms of clinical practice, we observed that food, particularly high-fat meals, significantly reduced the AUC of OXO, indicating the need for careful dose adjustment strategies. Clinicians may need to tailor the timing or dosage of S-1 administration according to the patients’ dietary habits, optimizing therapeutic efficacy while minimizing gastrointestinal side effects. Our findings underscore the importance of patient education, particularly regarding the necessity of taking S-1 on an empty stomach. Regarding renal function monitoring, since CDHP is primarily excreted via the kidneys, it is critical to monitor renal function to prevent potential drug accumulation and toxicity, particularly in patients with compromised renal function, who may require dose adjustments. From a global application perspective, the polymorphism of CYP2A6 suggests that Asian populations may experience higher FT exposure compared to Europeans, highlighting the need for dose adjustments based on CYP2A6 genotyping in different populations. This research provides valuable data for regulatory bodies evaluating generic S-1 formulations, demonstrating their BE and safety relative to branded versions. Such evidence may enhance the accessibility and affordability of S-1 for patients worldwide.

## Conclusion

This study was designed as a multicenter, randomized, open-label, single-dose, two-cycle crossover investigation aimed at evaluating the BE and safety of two formulations of S-1 in Chinese cancer patients, both under fasting and fed conditions. The evaluation of BE was based on the primary PK parameters. The results showed that C_max_, AUC_0-t_, and AUC_0- ∞_ all conformed to the established criteria for BE, supporting the BE of the two formulations. The two formulations of S-1 were safe in Chinese cancer patients under fasting and fed states.

## Data Availability

The original contributions presented in the study are included in the article/Supplementary Material; further inquiries can be directed to the corresponding authors.
